# The Impact of FGFR3 Alterations on the Tumor Microenvironment and the Efficacy of Immune Checkpoint Inhibitors in Bladder Cancer

**DOI:** 10.1186/s12943-023-01897-6

**Published:** 2023-11-18

**Authors:** Kazumasa Komura, Kensuke Hirosuna, Satoshi Tokushige, Takuya Tsujino, Kazuki Nishimura, Mitsuaki Ishida, Takuo Hayashi, Ayako Ura, Takaya Ohno, Shogo Yamazaki, Keita Nakamori, Shoko Kinoshita, Ryoichi Maenosono, Masahiko Ajiro, Yuki Yoshikawa, Tomoaki Takai, Takeshi Tsutsumi, Kohei Taniguchi, Tomohito Tanaka, Kiyoshi Takahara, Tsuyoshi Konuma, Teruo Inamoto, Yoshinobu Hirose, Fumihito Ono, Yuichi Shiraishi, Akihide Yoshimi, Haruhito Azuma

**Affiliations:** 1 Department of Urology, Osaka Medical and Pharmaceutical University, 2-7 Daigaku-Machi, Takatsuki City, Osaka, 569-8686 Japan; 2 Division of Translational Research, Osaka Medical and Pharmaceutical University, 2-7 Daigaku-Machi, Takatsuki City, Osaka, 569-8686 Japan; 3 Department of Regenerative Science, Okayama University Graduate School of Medicine, Dentistry and Pharmaceutical Sciences, 2-5-1 Shikata-Cho Kitaku, Okayama City, Okayama, 700-8558 Japan; 4 Division of Cancer RNA Research, National Cancer Center Research Institute, 5-1-1 Tsukiji, Chuo-Ku, Tokyo, 104-0045 Japan; 5 Department of Pathology, Osaka Medical and Pharmaceutical University, 2-7 Daigaku-Machi, Takatsuki City, Osaka, 569-8686 Japan; 6 Department of Human Pathology, Juntendo University Graduate School of Medicine, 2-1-1 Hongo, Bunkyo-Ku, Tokyo, 113-8421 Japan; 7 Department of Urology, Fujita-Health University School of Medicine, Toyoake City, 1-98 Dengakugakubo, KutsukakeAichi, 470-1192 Japan; 8 Graduate School of Medical Life Science, Yokohama City University, 1-7-29 Suehiro-Cho, Tsurumiku-Ku, Yokohama, Kanagawa 230-0045 Japan; 9 Division of Genome Analysis Platform Development, National Cancer Center Research Institute, 5-1-1 Tsukiji, Chuo-Ku, Tokyo, 104-0045 Japan

**Keywords:** Bladder cancer, Fibroblast growth factor receptor, Mutation, Fusion, Tumor microenvironment, Immune checkpoint inhibitor, Molecular subtypes

## Abstract

**Background:**

Currently, only limited knowledge is available regarding the phenotypic association between fibroblast growth factor receptor 3 (FGFR3) alterations and the tumor microenvironment (TME) in bladder cancer (BLCA).

**Methods:**

A multi-omics analysis on 389 BLCA and 35 adjacent normal tissues from a cohort of OMPU-NCC Consortium Japan was retrospectively performed by integrating the whole-exome and RNA-sequence dataset and clinicopathological record. A median follow-up duration of all BLCA cohort was 31 months.

**Results:**

*FGFR3* alterations (aFGFR3), including recurrent mutations and fusions, accounted for 44% of non-muscle invasive bladder cancer (NMIBC) and 15% of muscle-invasive bladder cancer (MIBC). Within MIBC, the consensus subtypes LumP was significantly more prevalent in aFGFR3, whereas the Ba/Sq subtype exhibited similarity between intact FGFR3 (iFGFR3) and aFGFR3 cases. We revealed that basal markers were significantly increased in MIBC/aFGFR3 compared to MIBC/iFGFR3. Transcriptome analysis highlighted TIM3 as the most upregulated immune-related gene in iFGFR3, with differential immune cell compositions observed between iFGFR3 and aFGFR3. Using EcoTyper, TME heterogeneity was discerned even within aFGFR cases, suggesting potential variations in the response to checkpoint inhibitors (CPIs). Among 72 patients treated with CPIs, the objective response rate (ORR) was comparable between iFGFR3 and aFGFR3 (20% vs 31%; *p* = 0.467). Strikingly, a significantly higher ORR was noted in LumP/aFGFR3 compared to LumP/iFGFR3 (50% vs 5%; *p* = 0.022). This trend was validated using data from the IMvigor210 trial. Additionally, several immune-related genes, including IDO1, CCL24, IL1RL1, LGALS4, and NCAM (CD56) were upregulated in LumP/iFGFR3 compared to LumP/aFGFR3 cases.

**Conclusions:**

Differential pathways influenced by aFGFR3 were observed between NMIBC and MIBC, highlighting the upregulation of both luminal and basal markers in MIBC/aFGFR3. Heterogeneous TME was identified within MIBC/aFGFR3, leading to differential outcomes for CPIs. Specifically, a favorable ORR in LumP/aFGFR3 and a poor ORR in LumP/iFGFR3 were observed. We propose TIM3 as a potential target for iFGFR3 (ORR: 20%) and several immune checkpoint genes, including IDO1 and CCL24, for LumP/iFGFR3 (ORR: 5%), indicating promising avenues for precision immunotherapy for BLCA.

**Supplementary Information:**

The online version contains supplementary material available at 10.1186/s12943-023-01897-6.

## Introduction

BLCA poses a significant global health challenge, ranking as the fourth most common cancer and the eighth leading cause of cancer-related deaths in men [1]. The introduction of immune checkpoint inhibitors (CPIs) in BLCA treatment, sanctioned by the Food and Drug Administration (FDA) in 2017, marked a pivotal shift in therapeutic strategies [2]. However, the efficacy of CPIs remains limited, with the majority of patients showing minimal or no response. Patients with progressive disease (PD) at their best overall response accounted for 48.5% with no survival benefit compared to the second-line chemotherapy [3]. Therefore, understanding the molecular intricacies governing CPI response and exploring innovative methods to enhance CPI effectiveness are imperative.

Genetic alterations in fibroblast growth factor receptor 3 (FGFR3) are frequently identified in BLCA [4, 5]. Previous studies have linked *FGFR3* alterations (aFGFR3) to luminal papillary tumors characterized by diminished T-cell infiltrations [6]. While initial assumptions suggested reduced sensitivity of aFGFR3 tumors to CPIs, recent research contradicts this notion, demonstrating comparable CPI effects between aFGFR3 and intact FGFR3 (iFGFR3) BLCA cases [7, 8]. Consequently, exploring the tumor microenvironment (TME) in the context of *FGFR3* status emerges as a promising avenue for novel therapeutic interventions. To address this, we conducted a comprehensive multi-omics analysis encompassing 389 BLCA cases and 35 adjacent normal tissues, aiming to unravel the intricate relationship between aFGFR3 and the TME.

## Results

### FGFR Alterations in NMIBC and MIBC

**Fig. 1:**
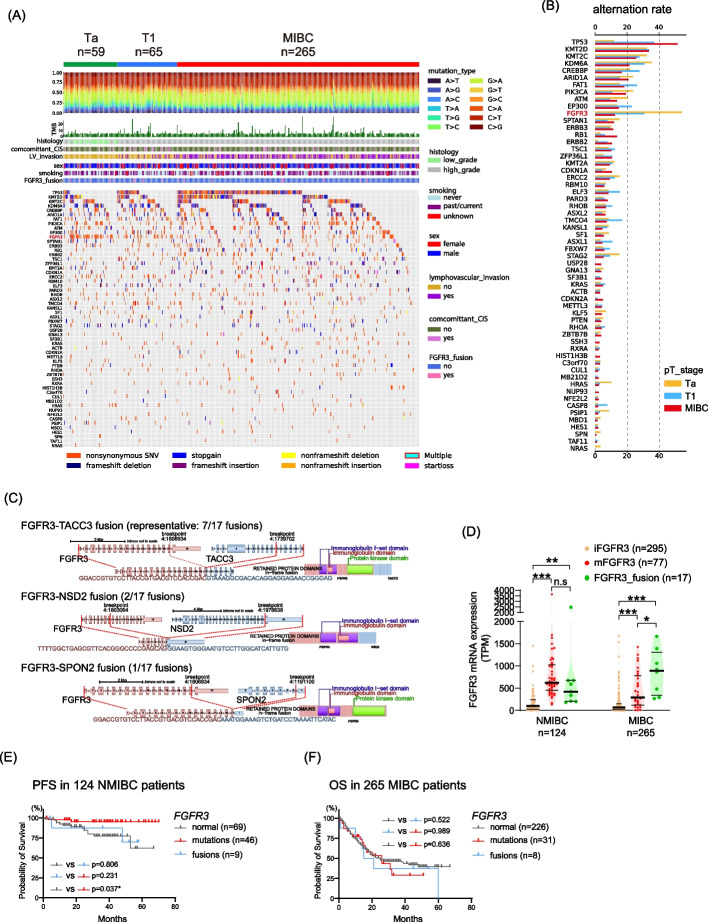
FGFR Alterations in NMIBC and MIBC. **A** Mutation landscape of 58 significantly mutated genes defined by the TCGA publication [9] in 389 bladder cancer (BLCA) samples from the OMPU-NCC cohort. The patients were classified into pTa (*n* = 59), pT1 (*n* = 65), and ≥ pT2 (*n* = 265, MIBC: muscle-invasive bladder cancer). **B** Recurrent mutation rate of 58 significantly mutated genes according to pathological T stages. **C** Schematic of the *FGFR3* fusions identified in our cohort. FGFR3-TACC3 fusions were found in 14 of 289 patients, and the most frequent pattern (7 of 11) is shown. *NSD2* and *SPON2* are newly identified fusion partners. **D** FGFR3 mRNA expression levels according to the *FGFR3* alterations. The difference was assessed by the Mann–Whitney U test; *p* < 0.05*, *p* < 0.001**, *p* < 0.0001***. **E**, **F** Kaplan–Meier curves demonstrating progression-free survival (PFS) in non-muscle-invasive bladder cancer (NMIBC) (**E**) and overall survival (OS) in MIBC (**F**). A log-rank test was used to assess the survival difference between the two groups; *p* < 0.05*

The cohort in the present study included 124 non-muscle invasive bladder cancer (NMIBC: 5-year overall survival (OS) rate: 83%) and 265 muscle-invasive bladder cancer (MIBC: 5-year OS rate: 35%) with a median follow-up of 36 and 22 months, respectively (Supplementary Fig. 1A). We first assessed mRNA expression levels of FGFR family genes in the cohort. *FGFR3* is actively transcribed among the FGFR families in the bladder, especially in BLCA (Supplementary Fig. 1B). *FGFR3* was the most frequently mutated gene (81/389; 21%) among the FGFR families including *FGFR1* (19/389; 5%), *FGFR2* (8/389; 2%), and *FGFR4* (12/389; 3%) (Supplementary Fig. 1C). A previous TCGA publication defined 58 significantly mutated genes (SMGs) in BLCA [9], which was largely recapitulated in our cohort including the clinicopathological characteristics (Fig. 1A, B).

To delineate the allelic difference in *FGFR3* among ethnicity, we sought to assess whether there is a specific allelic variant in the germline for the Asian population. GnomADv3.0, an integrative germline dataset of 71,702 individuals (mostly Western population), was utilized for the control [10]. We referred the Asian germline dataset (jMorp-14KJPN) [11] and identified five significantly enriched non-synonymous single nucleotide polymorphisms (SNPs) on the *FGFR3* gene locus that are specific to the Asian population (Q29H, G65R, L164V, T450M, and A720S) (Supplementary Fig. 1D). However, these SNPs were not enriched in BLCA samples (Supplementary Table 1). Compared to BLCA with iFGFR3, FGFR3 mRNA expression level was significantly upregulated in patients with recurrent *FGFR3* mutations but not in patients with the SNPs (Supplementary Fig. 1E). There seemed to be no survival difference based on the *FGFR3* status (Supplementary Fig. 1F), indicating no clinical implication of these Asian-specific SNPs in *FGFR3*.

FGFR3 mutations was predominantly observed in cases with lower malignant properties such as NMIBC (pTa: 51%, pT1: 29%, more than pT2: 12%), low grade and negative lymph vascular invasions (Table 1). Regarding the mutational alleles, the TCGA publication, which only consists of MIBC samples, reports S249C and Y373C as the top two frequent *FGFR3* mutations in BLCA (Supplementary Fig. 2A). We noted that recurrent K650E and T757P nonsynonymous mutations at the kinase domain (KD) were frequently observed in MIBC in our cohort compared to the TCGA cohort (Supplementary Fig. 2B). Although we examined the prognosis of five MIBC cases with mutation at KD (three in K650E and two in T757P), there was no difference in OS compared to that in cases with other *FGFR3* alterations (Supplementary Fig. 2C). Interestingly, we found that *FGFR3* mutations at KD were more prevalent in MIBC than in NMIBC cases (*p* = 0.021) (Supplementary Fig. 2D).

**Table 1:** Clinicopathological characteristics in 389 BLCA patients according to the *FGFR3* status at the collection of biospecimens

		*FGFR3* mutations (nonsynonymous/indels)			*FGFR3* fusion		
Variables	*n* = 389	mutation -*n* = 308 (79%)	mutation + *n* = 81 (21%)	*p* value	fusion -*n* = 372 (96%)	fusion + *n* = 17 (4%)	*p* value
Sex (%)							
male	306 (79)	243 (79)	63 (78)		291 (78)	15 (88)	
female	83 (21)	65 (21)	18 (22)	0.83	81 (22)	2 (12)	0.29
Age (mean ± SD)							
	70 ± 11.2	69 ± 11.1	70 ± 11.5	0.52	70 ± 11.2	69 ± 10.6	0.82
Smoking history (%)							
never	132 (34)	102 (33)	30 (37)		127 (34)	5 (29)	
past/current	242 (62)	193 (63)	49 (60)		231 (62)	11 (65)	
unnown	15 (4)	13 (4)	2 (3)	0.64	14 (4)	1 (6)	0.86
Clinical Stage (%)							
cN0M0	315 (81)	244 (79)	71 (87)		301 (81)	14 (82)	
cN1M0	51 (13)	44 (14)	7 (9)		50 (13)	1 (6)	
cNxM1	23 (6)	20 (7)	3 (4)	0.2	21 (6)	2 (12)	0.43
Muscle invasion (%)							
NMIBC	124 (32)	75 (24)	49 (61)		115 (31)	9 (53)	
MIBC	265 (68)	233 (76)	32 (39)	< 0.001*	257 (69)	8 (47)	0.06
Histological variants (%)							
no	345 (89)	270 (88)	75 (93)		328 (88)	17 (100)	
yes	44 (11)	38 (12)	6 (7)	0.19	44 (12)	0 (0.0)	0.04*
Concomittant CIS (%)							
no	333 (86)	258 (84)	75 (93)		320 (86)	13 (76)	
yes	56 (14)	50 (16)	6 (7)	0.03*	52 (14)	4 (24)	0.31
Pathological grade (WHO2004)							
low	40 (10)	19 (6)	21 (26)		37 (10)	3 (18)	
high	349 (90)	289 (94)	60 (74)	< 0.001*	335 (90)	14 (82)	0.35
Lymphovascular invasion							
no	179 (46)	121 (39)	58 (72)		169 (45)	10 (59)	
yes	210 (54)	187 (61)	23 (28)	< 0.001*	203 (55)	7 (41)	0.28
Median follow-up period							
(months: [IQR])	31 [15, 44]	29 [14, 42]	38 [20, 45]		31 [15, 42]	47 [23, 55]	

*BLCA* Bladder cancer, *FGFR3* Fibroblast growth factor receptor 3, *TACC3* Transforming acidic coiled-coil containing protein 3, *SD* Standard deviation, *NMIBC* Non-muscle invasive bladder cancer, *MIBC* Muscle invasive bladder cancer, *CIS* Carcinoma in situ, *WHO* World Health Organization, *BCG* Bacille de Calmette et Guérin, *IQR* Interquartile range

^*^Denotes *p* < 0.05

FGFR3 mRNA expression levels were consistently upregulated in aFGFR3 compared to iFGFR3, regardless of the mutation sites (Supplementary Fig. 2E). The present study exhibited a frequency of 4% (17/389) for *FGFR3* fusions (Table 1), including novel fusion partners (*NSD2* and *SPON2*) (Fig. 1C). No histological variant was observed in cases with FGFR3 fusions (Table 1). The KD located at the C-terminus of FGFR3 has been retained in 13 of 17 (77%) fusions, and the mRNA expression level was significantly upregulated in cases with *FGFR3* fusions compared to cases with iFGFR3 (Supplementary Fig. 2F). Upon stratifying our cohort into NMIBC and MIBC categories, FGFR3 mRNA expression levels were significantly higher in aFGFR3 cases than in iFGFR3 cases in both NMIBC and MIBC (Fig. 1D), with the highest median mRNA expression levels observed in MIBC patients with *FGFR3* fusions. This finding underscores the clinical importance of detecting *FGFR3* fusions, alongside mutations, in advanced MIBC patients and accentuates the importance of considering recently approved FGFR3 inhibitors [12]. The FGFR3 protein expression levels were increased in aFGFR3 compared to iFGFR3 cases (Supplementary Fig. 2G, H). We investigated the progression free survival (PFS) of 124 NMIBC patients (Fig. 1E). Patients with recurrent *FGFR3* mutations showed a significantly better PFS compared to those with iFGFR3 (*p* = 0.037). However, this distinction was not evident in patients with *FGFR3* fusions (*p* = 0.806). In the context of OS among 265 MIBC patients, no significant differences were observed based on *FGFR3* status (Fig. 1F).

### The Association between FGFR3 alteration and molecular Subtypes

**Fig. 2:**
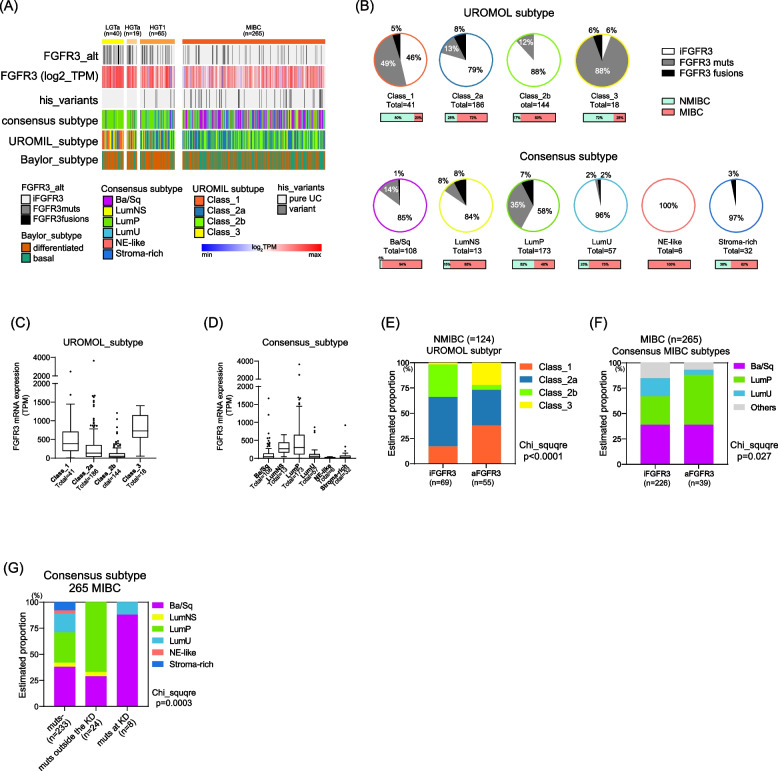
Association between FGFR3 Alteration and Molecular Subtypes. **A** Summary of *FGFR3* alterations, FGFR3 mRNA expression, histological variant, consensus MIBC subtypes [6], UROMOL NMIBC subtypes [13], and Baylor college [14]. **B** Pie chart of *FGFR3* alterations including recurrent mutations and fusions in 389 BLCA cases. **C** FGFR3 mRNA expression levels according to UROMOL subtypes. **D** FGFR3 mRNA expression levels according to consensus MIBC subtypes. **E** Estimated proportion of UROMOL subtypes in 124 NMIBC cases. **F** Estimated proportion of consensus MIBC subtypes in 265 MIBC cases. **G** Estimated proportion of consensus MIBC subtypes in 265 MIBC cases categorized based on *FGFR3* mutational status (KD: kinase domain; note that seven cases with *FGFR3* fusions were classified into mut- group)

We have adopted the established consensus MIBC subtype [6], the UROMOL subtype for NMIBC [13], and Baylor college subtype [14] (Fig. 2A and Supplementary Fig. 3A). As expected, distribution of subtypes significantly differed between NMIBC and MIBC across these three subtyping systems (Consensus MIBC: *p* < 0.0001, UROMOL: *p* < 0.0001, and Baylor: *p* < 0.0001) (Supplementary Fig. 3B-3D). In the overall cohort (*n* = 389), *FGFR3* alterations were enriched in class_1 (54%) and class_3 (94%) for the UROMOL subtype and LumP (42%) for consensus MIBC subtype (Fig. 2B and Supplementary Fig. 3E). An elevated FGFR3 mRNA expression level was confirmed within these molecular subtypes (Fig. 2C, D and Supplementary Fig. 3F).

We further stratified the cohort into NMIBC (*n* = 124) and MIBC (*n* = 265) cohorts. In NMIBC, the UROMOL subtype showed a significant increase in class_1 and class_3 subtypes among aFGFR3 cases (*p *< 0.0001) (Fig. 2E). In MIBC, the consensus MIBC subtypes exhibited a significantly higher prevalence of the LumP subtype in aFGFR3 cases (49% vs 28%) (Fig. 2F). Interestingly, the proportion of Ba/Sq subtype in MIBC cases was similar between iFGFR3 and aFGFR3 (39% vs 38%). Other subtypes, such as LumU, appeared to decrease in aFGFR3 cases in place of an increase in LumP. Considering the enrichment of *FGFR3* mutations at KD in MIBC cases (Supplementary Fig. 2D), we further assessed their association with molecular subtypes. Mutations at KD were notably inclined towards the Ba/Sq subtype (seven of eight: 88%), contrasting with other mutations (24 cases: 67% in LumP and 29% in Ba/Sq subtypes) (Fig. 2G). These findings illuminate the intricate interplay between aFGFR3 and BLCA subtypes.

### Different Pathways Modified by aFGFR3 between NMIBC and MIBC

**Fig. 3:**
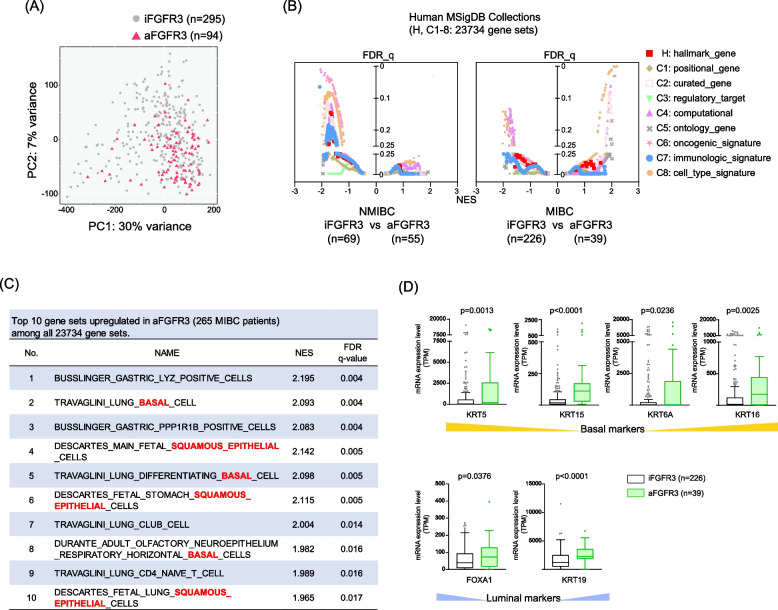
Different Pathways Modified by aFGFR3 between NMIBC and MIBC. **A** Principal component analysis for whole transcriptome data in iFGFR3 and aFGFR3. **B** Gene set enrichment analysis plotting all human MSigDB collections (Hallmark, C1-8: 23,734 gene sets) by false discovery rate q-value (FDR-q) and normalized enrichment score (NES). The analysis was performed separately in NMIBC (left panel) and MIBC (right panel). **C** Top 10 gene sets upregulated in MIBC/aFGFR3 (*n* = 39) compared to MIBC/iFGFR3 (*n* = 226). **D** mRNA expression levels in putative basal markers (KRT5, 15, 6A, and 16) and luminal markers (FOXA1 and KRT19). The difference in the expression level was assessed by the Mann–Whitney U test

Principal component analysis of the whole transcriptome exhibited the delineation of aFGFR3 (Fig. 3A). Thus, we examined gene set enrichment analysis (GSEA) for all human collections (H, C1-C8: 23,734 gene sets) (Supplementary Fig. 3G). There was no gene set showing a false discovery rate (FDR) of < 0.25 in C7 (immunologic_signature_gene_sets), whereas H (hallmark_gene_sets) represented epithelial-mesenchymal transition (EMT) pathway as the most down-regulated pathways in aFGFR3 (Supplementary Fig. 3H, Supplementary Table 2).

To further delve into the insight of affected pathways in aFGFR3, we re-ran GSEA after separating the cohort into NMIBC (*n* = 124) and MIBC (*n *= 265) groups. Interestingly, the pathways exhibiting significant difference between iFGFR3 and aFGFR3 cases (FDR_q value < 0.25) were notably distinct in NMIBC and MIBC (Fig. 3B). In NMIBC (69: iFGFR3 vs 55: aFGFR3), only one (1/23,734 gene sets) pathway (C2: LINDGREN_BLADDER_CANCER_CLUSTER_3_DN) was significantly upregulated in aFGFR3 cases, while 3,630 pathways (C1: 6, C2: 940, C3: 0, C4: 133, C5: 1372, C6: 120, C7: 596, C8: 457, H: 6) were significantly downregulated in aFGFR3 cases. In stark contrast, MIBC (226: iFGFR3 vs 39: aFGFR3) exhibited significant elevation in 140 pathways (C1: 2, C2: 45, C3: 0, C4: 46, C5: 3, C6: 0, C7: 0, C8: 44, H: 0) and downregulation in 95 pathways (C1: 0, C2: 0, C3: 0, C4: 44, C5: 0, C6: 5, C7: 0, C8: 46, H: 0) in aFGFR3 cases (Supplementary Table 3). Importantly, we discovered that the top 10 upregulated pathways in MIBC with aFGFR3 include pathways associated with “basal/squamous epithelial” characteristics (Fig. 3C). This observation was further substantiated by the significant increase in known basal markers such as KRT5, KRT15, KRT6A, and KRT16, as well as typical luminal markers like FOXA1 and KRT19 (Fig. 3D). These findings collectively suggest the substantial variation in pathways influenced by aFGFR3 between NMIBC and MIBC. Moreover, in addition to the previous findings indicating that aFGFR3 is associated with luminal subtypes [6], our results reveal that aFGFR3 in MIBC can influence both epithelial subtypes, including the basal/squamous type.

### Immune checkpoint genes and immune-related cell compositions in aFGFR3

**Fig. 4:**
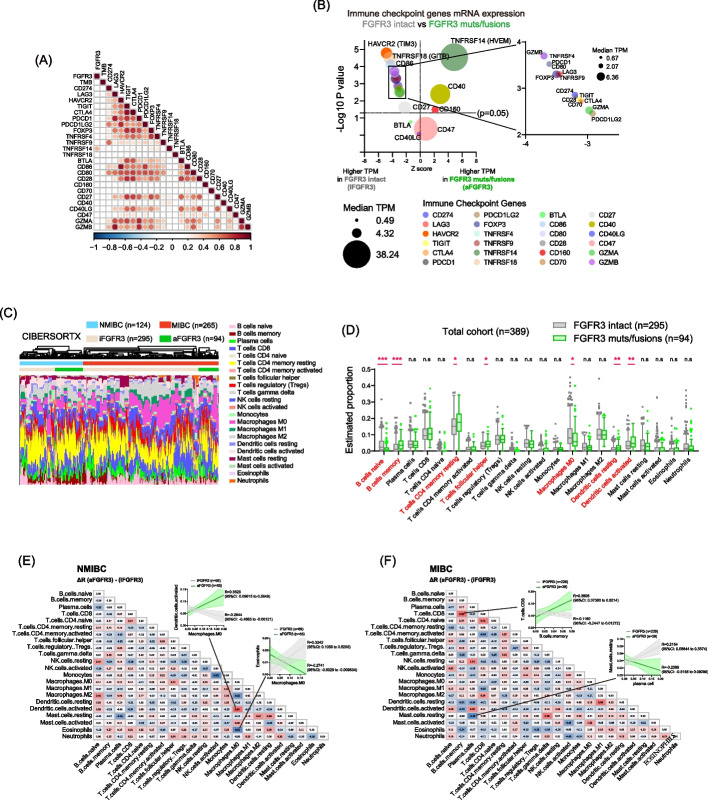
Immune Checkpoint Genes and Immune-Related Cell Compositions in aFGFR3. **A** Spearman rank correlation among *FGFR3* status, tumor mutation burden (TMB), and immune-checkpoint genes in 389 BLCA. **B** Bubble plots of difference in the immune checkpoint genes expression levels according to FGFR3 status. **C** CIBERSORTx analysis estimating composition of immune-related cells in 389 BLCA samples [15]. **D** The estimated proportion of each immune cell type from CIBERSORTx in iFGFR3 and aFGFR3 (Mann–Whitney U test; *p* < 0.05*, *p* < 0.001**, *p* < 0.0001***, n.s: non-significant). **E** The difference in correlation coefficient among estimated proportions of immune-related cells between NMIBC/iFGFR3 (*n* = 69) and NMIBC/aFGFR3 (*n* = 55). **F** The difference in correlation coefficient among estimated proportions of immune-related cells between MIBC/iFGFR3 (*n* = 226) and MIBC/aFGFR3 (*n* = 39)

We next examined TME according to the FGFR3 status. First, PD-L1 expression as evaluated by the combined positive score (CPS) was positively correlated with CD274 mRNA expression (Supplementary Fig. 4A-B), and the CPS seemed to be lower in aFGFR3 than in iFGFR3 (*p* = 0.07) (Supplementary Fig. 4C-D). Similar to the previous studies, CD8 + T-cell counts in the specimens seemed lower in aFGFR3 (*p* = 0.088) (Supplementary Fig. 4E-F). We explored the correlation between *FGFR3* status and expression levels of putative immune checkpoint genes in 389 BLCA (Supplementary Fig. 4G, Supplementary Table 4). There was a positive correlation among immune checkpoint genes, whereas the correlation of these genes with *FGFR3* was modest (Fig. 4A). We analyzed the potential candidates that are differentially expressed between iFGFR3 and aFGFR3 by analyzing the expression of the immune checkpoint genes, and identified that T-cell exhaustion markers, including TIM3, were most upregulated in iFGFR3, whereas HVEM and dendritic cell marker such as CD40 were increased in aFGFR3 (Fig. 4B, Supplementary Fig. 4H).

We next estimated immune cell composition by using CIBERSORTx, a digital cytometry from bulk tissues [15] (Supplementary Table 5), and revealed distinct infiltration patterns of various immune cell types in BLCA according to *FGFR3* status, including B naïve cells, B memory cells, T-CD4 + memory resting cells, T-follicular helper cells, M0 macrophages, dendritic cell proportions (Fig. 4C-D). We also confirmed that the actual cell count (CD8 and FOXP3) in the specimens and the estimated proportion was significantly correlated (Supplementary Fig. 5A).

Since the comprehensive GSEA showed differential pathways influenced by aFGFR3 between NMIBC and MIBC (Fig. 3B), we compared immune cell composition based on *FGFR3* status within NMIBC (*n* = 124) and MIBC (*n* = 265) (Supplementary Fig. 5B-C). The correlation between immune cell proportions varied between iFGFR3 and aFGFR3 in NMIBC and MIBC cases (Supplementary Fig. 5D-E). Specifically, in NMIBC, we observed a negative correlation between the estimated populations of M0 macrophages and activated dendritic cells in iFGFR3 (*r* = -0.2844, 95% CI: -0.4883 to -0.05121), while a positive association between these cells was evident in aFGFR3 (*r* = 0.3525, 95% CI: 0.09615 to 0.5649) (Fig. 4E). In MIBC, a negative correlation between the estimated populations of CD8 T cells and memory B cells was found in iFGFR3 (*r *= -0.1180, 95% CI: -0.2447 to -0.01272), whereas a positive association between these cells was observed in aFGFR3 (*r* = 0.3805, 95% CI: 0.07380 to 0.6214) (Fig. 4F). These data collectively indicate differential immune cell compositions between iFGFR3 and aFGFR3, highlighting the distinct biological impact of aFGFR3 on the TME, which further varies between NMIBC and MIBC.

### TME Heterogeneity in aFGFR3

**Fig. 5:**
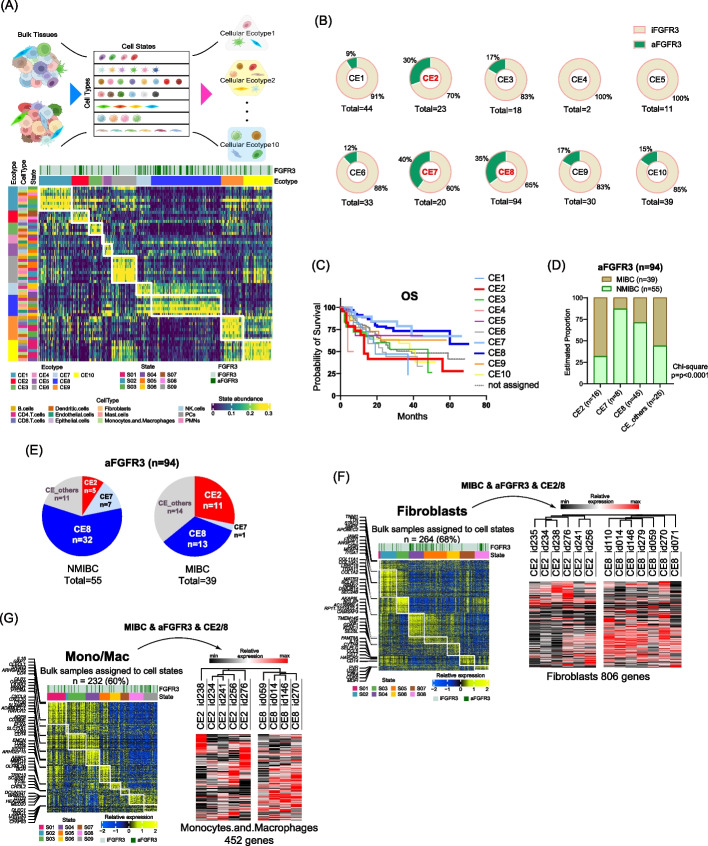
TME Heterogeneity in aFGFR3. **A** EcoTyper analysis identifying cell states from bulk expression data, which covers 12 major cell lineages, including immune-related cells, fibroblasts, endothelial cells, and epithelial cells [16]. A total of 69 transcriptional cell states were identified, and an analysis on the cell-state co-occurrence patterns offered ten clinically distinct multicellular communities known as Cellular Ecotypes (CE1-10). CEs had been shown to correlate with OS (shorter in CE1 and longer in CE10) and treatment response to checkpoint inhibitors (CPIs) in various types of cancer data sets. Note that In CE2 and CE8, different cell states were depicted in specific cell types, including fibroblasts, epithelial cells, and mono/macrophages. **B** Pie charts showing the proportion of patients with aFGFR3 in each CE. **C** Kaplan–Meier curves of OS according to the cell ecotypes (CE1-10). **D** Estimated proportion of NMIBC/aFGFR3 (*n* = 55) and MIBC/aFGFR3 (*n* = 39) among CE2, CE7, CE8, and other CEs. **E** Distribution of CE2, CE7, CE8, and other CEs in NMIBC/aFGFR3 (*n* = 55) and MIBC/aFGFR3 (*n* = 39). **F** Cell state for the fibroblasts was defined by EcoTyper in 264/389 (68%) of the present cohort. Among them, we compared the mRNA expression of 806 genes determining the state of fibroblasts in CE2 and CE8 for MIBC/aFGFR3 patients. **G** Cell state for the monocytes/macrophages was defined by EcoTyper in 232/389 (60%) of the present cohort. Among them, we compared the mRNA expression of 452 genes determining the state of monocytes/macrophages in CE2 and CE8 for MIBC/aFGFR3 patients

To further explore the TME differences according to *FGFR3* status, we employed the EcoTyper (Fig. 5A) [16]. EcoTyper is a machine learning pipeline for identifying cell states from bulk expression data, which covers 12 major cell lineages, including immune-related cells, fibroblasts, endothelial cells, and epithelial cells (Supplementary Fig. 5F). This methodology delineated 69 transcriptionally distinct cell states, unveiling ten clinically relevant multicellular communities known as Cellular Ecotypes (CE1-10). These ecotypes showed significant correlations with OS (shorter in CE1 and longer in CE10) and response to CPIs across various cancer datasets.

In our BLCA cohort, we noticed an enrichment of aFGFR3 (mutations/fusions) in specific CEs (CE2, CE7, and CE8) (Fig. 5B). Kaplan–Meier curves for each CE exhibited distinct OS favoring CE7 and CE8, whereas CE2 showed the worst OS with a median of 13 months (Fig. 5C). Upon further analysis of the NMIBC (*n* = 124) and MIBC (*n* = 265) cases, we found that CE2 predominantly comprised MIBC patients, whereas CE7 and CE8 were associated with NMIBC patients in aFGFR3 (*n* = 94) (Fig. 5D).

To explore the association between the TME in aFGFR3 and the response to CPIs, we focused on MIBC patients within CE2 and CE8. Of 39 MIBC/aFGFR3 cases, 11 (28%) and 13 (33%) were defined as CE2 and CE8, respectively (Fig. 5E). In these two CEs, different cell states were depicted in specific cell types, including fibroblasts, epithelial cells, and mono/macrophages (Fig. 5A). By extracting the expression matrix from the EcoTyper pipeline, we identified unique expression patterns in fibroblasts 806 genes (Fig. 5F) and mono/macrophages 452 genes (Fig. 5G) between CE2/aFGFR3 and CE8/aFGFR3 cases. These data collectively suggest the remarkable TME heterogeneity within the aFGFR3 subgroup, potentially influencing the efficacy of CPIs.

### Response of CPIs according to molecular subtypes and FGFR3 status

**Fig. 6:**
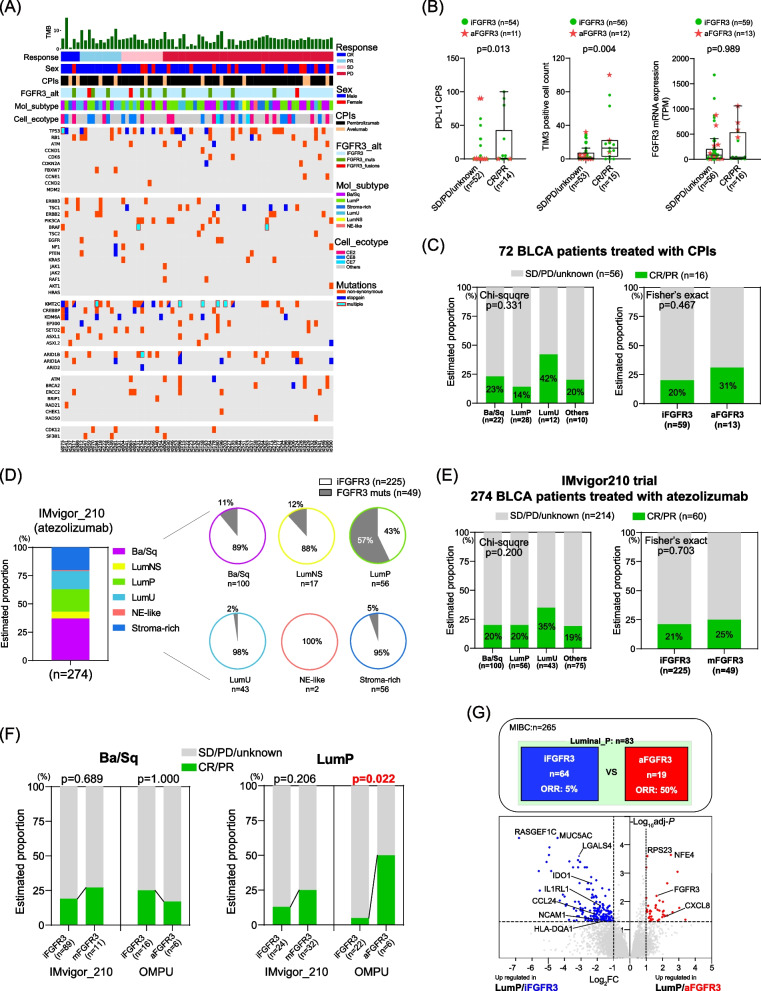
Response of CPIs According to Molecular Subtypes and FGFR3 Status. **A** Of 389 BLCA patients, 72 were treated with CPIs including PD-1 inhibitor pembrolizumab (*n* = 60) and PD-L1 inhibitor avelumab (*n* = 12). Oncoprint sorted by the treatment response in those 72 patients is shown. **B** PD-L1 combined positive score (CPS), Cell count of TIM3 + cells in high power field, and FGFR3 mRNA expression were compared according to the response to CPIs. The difference was assessed by the Mann–Whitney U test. **C** The Objective response rate (ORR) in 72 patients treated with CPIs according to consensus subtypes (left panel) and *FGFR3* alterations (right panel). **D** The estimated proportion of consensus subtypes in the IMvigor210 trial (PD-L1 inhibitor atezolizumab in patients with metastatic urothelial carcinoma) [17]. Pie charts show the proportion of *FGFR3* mutations (not included for *FGFR3* fusions) among the subtypes. **E** The ORR in 274 patients treated with atezolizumab according to consensus subtypes (left panel) and *FGFR3* mutations (right panel). **F** The ORR in the IMvigor210 trial (*n* = 274) and the present cohort (OMPU: *n* = 72) treated with CPIs in Ba/Sq subtype (left panel) and LumP subtype (right panel). Fisher’s exact test was performed to assess the difference of the ORR according to *FGFR3* status. Note that the data from IMvigor210 does not include the information on *FGFR3* fusions. **G** Differentially expressed gene (DEG) analysis between MIBC/LumP/iFGFR3 (*n* = 64) and MIBC/LumP/aFGFR3 (*n* = 19) in the present cohort

In the present cohort, 72 of 389 patients were treated with CPIs (pembrolizumab: 60 patients and avelumab: 12 patients) (Fig. 6A). The objective response rate (ORR) was 22% (pembrolizumab: 20% and avelumab 33%) (Supplementary Fig. 6A). We first assessed the ORR according to CEs from EcoTyper (Supplementary Fig. 6B). In line with the original report [16], a favorable ORR in CE10 (40% in 10 cases) and a poor ORR in CE6 (0% in 8 cases) were confirmed. The ORR in the top two allocated ecotypes were 21% in CE2 (*n* = 14) and 29% in CE8 (*n* = 14). Patients achieving CR/PR exhibited significantly higher PD-L1 CPS (*p* = 0.013) and TIM3 positive cells (*p* = 0.004) than those with SD/PD/unknown response (Fig. 6B). Tumor mutation burden (TMB) was also positively correlated with PD-L1 CPS (*p* = 0.021) and TIM3 positive cells (*p* = 0.009) (Supplementary Fig. 6C).

Regarding molecular subtypes, the ORR was 23%, 14%, 42%, and 20% in Ba/Sq, LumP, LumU, and other subtypes, respectively (*p* = 0.331) (Fig. 6C). The ORR to CPIs was comparable in aFGFR3 compared to iFGFR3 (31% vs 20%; *p* = 0.467). To validate our result, we analyzed the data from the IMvigor 210 trial [17], exploring the PD-L1 inhibitor atezolizumab in patients with metastatic urothelial carcinoma (UC) using RNA-seq and hybrid capture-based next-generation sequencing for 274 patients. Despite lacking information on *FGFR3* fusions, we assessed the distribution of consensus MIBC molecular subtypes and *FGFR3* mutations (Fig. 6D). Of 49 patients with *FGFR3* mutations, 11 (23%), 32 (65%), 1 (2%), and 5 (10%) were assigned to Ba/Sq, LumP, LumU, and other subtypes, respectively, exhibiting a higher LumP proportion in aFGFR3 than in iFGFR3 (*p* < 0.0001) (Supplementary Fig. 6D). LumU was rarely observed in aFGFR3, which was consistent with our cohort (Fig. 2F and Fig. 6D). The ORR in the IMvigor210 trial was 20%, 20%, 35%, and 19% in Ba/Sq, LumP, LumU, and other subtypes, respectively (*p* = 0.20) (Fig. 6E). Notably, the ORR in patients with *FGFR3* mutations (*n* = 49) was 25%, which was comparable to 21% in iFGFR3 cases (some of whom presumably harbored *FGFR3* fusions).

Our study revealed the remarkable heterogeneity within the TME even among the aFGFR3 cases. Thus, we stratified the cohort of 72 patients treated with CPIs based on molecular subtypes and *FGFR3* status (Fig. 6F and Supplementary Fig. 6E). In the Ba/Sq subtype, the ORR ranged from 17 to 27% regardless of *FGFR3* alterations, with no significant difference. However, the LumP subtype presented a striking contrast: a significantly higher ORR in aFGFR3 (50%) cases compared to a 5% in iFGFR3 cases in the present cohort (*p* = 0.022). This trend was also observed in the IMvigor 210 cohort with an ORR of 25% in FGFR3 mutations and 12% in iFGFR3. We performed gene expression analysis between LumP/iFGFR3 (*n* = 64) and LumP/aFGFR3 (*n* = 19) in the present cohort (Fig. 6G). Strikingly, several immune-related genes were significantly upregulated in LumP/iFGFR3 cases, including IDO1, CCL24, IL1RL1, LGALS4, and NCAM (CD56). These findings underscore the potential of these genes as promising targets for immunotherapy.

## Discussions

Several studies have suggested the association between *FGFR3* gene alternations and immune-related pathways [8, 18]. Sweis et al. reported that BLCA with *FGFR3* alterations were enriched in non–T-cell-inflamed tumors (poor T-cell infiltration) in the TCGA cohort and speculated the impaired response of CPIs in BLCA with aFGFR3 [18]. On the other hand, Wang et al. examined whether BLCA with *FGFR3* mutations shows a distinct clinical response to CPIs based on the data from two clinical trials: the IMVigor 210 trial [17] exploring the PD-L1 inhibitor atezolizumab in 274 patients with metastatic UC and the CheckMate 275 [19] exploring the PD-1 inhibitor nivolumab in 139 patients with metastatic UC [20]. They found no statistically significant difference in response rates of CPIs between patients with aFGFR3 versus iFGFR3. In their analysis, *FGFR3* mutant UC harbored a lower T-cell infiltration signature (putatively negative effect for CPIs), but also lower stromal/transforming growth factor-beta (TGFβ) signals (putatively positive effect for CPIs). They hypothesized that these factors counterbalance each other, resulting in a similar sensitivity to CPIs in both *FGFR3* mutant and wild-type UC. In the present study, unique in integration of whole transcriptome data, we delineated molecular subtypes and uncovered the distinct landscape of *FGFR3* alterations in the Asian population. Our comprehensive genetic and molecular analyses demonstrated that a number of immune checkpoint genes are differentially expressed between iFGFR3 and aFGFR3, and the deconvolution analysis revealed significant heterogeneity in CEs even within the aFGFR3 subgroup. While the clinical response to CPIs was comparable between iFGFR3 and aFGFR3, LumP/aFGFR3 cases displayed as high as 50% ORR in the cohort, whereas LumP/iFGFR3 cases showed the lowest ORR of 5%.

Upon comparing putative immune checkpoint genes between iFGFR3 and aFGFR3, we identified HAVCR2 (TIM3) as the most upregulated gene in iFGFR3 cases (Fig. 4B). We examined immunohistochemistry using TIM3 and assessed the correlation with the response to CPIs (Fig. 6B). TIM3 positive cases were significantly more prevalent in patients with objective response (*p* = 0.004). Cobolimab, a newly developed TIM3 inhibitor, is currently undergoing investigation in advanced solid tumor following the positive results from phase 1 AMBER trial (NCT02817633) [21]. Of 72 patients treated with CPIs in the present cohort, 47 iFGFR3 cases failed to achieve an objective response to CPIs. Among those 47 cases, the median TIM3 positive count at a high-power field was 1.5 (interquartile: 0–7 cells), which implies that TIM3 inhibition holds potential to benefit this particular patient population.

Furthermore, the gene expression analysis between LumP/aFGFR3 and LumP/iFGFR3 unveiled several genes correlated with the TME. For instance, IDO1, a crucial enzyme in the immune checkpoint pathway, acts as a metabolic enzyme converting the essential amino acid tryptophan into immunosuppressive metabolites, primarily via the kynurenine pathway [22]. It is recognized as an immune checkpoint molecule, which is produced by certain immune cells, and modulates immune responses by suppressing T and NK cells, aiding cancer cells in evading immune surveillance. Currently, inhibitors targeting IDO1 are being explored in clinical trials with the aim to undermine the immunosuppressive environment it creates, thereby enhancing anti-tumor immune responses and potentially improving the prognosis in various cancers [23].

The present study has several limitations. Its retrospective nature, utilizing BLCA clinical samples collected from the Osaka Medical and Pharmaceutical University biobank (OMPU biobank), might introduce biases related to regional and physician-related factors. Moreover, while our deconvolution approach provided valuable insights, a recent study raised concerns about its low sensitivity and specificity in predicting some specific cell populations, including CD4 T cells, macrophages, and monocytes [24]. These findings suggest caution in interpreting results derived from this method.

## Methods

### Clinical record and patient care

Clinical information was recorded at the time of biospecimen collection during tumor resection with either radical cystectomy or transurethral resection. No data points were excluded from the analyses unless is specified otherwise. The distribution of each factor was assessed using contingency tables and Fisher's exact or Chi-square analysis. Kolmogorov–Smirnov normality tests were performed to check the normal distribution of continuous variables, followed by conducting a student's t-test or one-way ANOVA to assess differences between variables. For variables with non-normal distributions, Mann–Whitney U or Kruskal–Wallis tests were performed to assess differences. Overall survival (OS) was calculated as the interval from tumor resection to the date of the last follow-up or death from any cause. For muscle-invasive bladder cancer (MIBC) patients, progression-free survival (PFS) was defined as the period from tumor resection to the diagnosis of radiographic or clinical progression. For non-muscle invasive bladder cancer (NMIBC) patients, PFS was defined as the period from tumor resection to recurrence with up-staging to MIBC or the development of metastasis. Kaplan–Meier survival analysis and log-rank tests were used to compare OS, cancer-specific survival (CSS), and PFS between the assigned groups. All statistical tests were two-tailed, with a threshold of *p* < 0.05 considered significant for statistical analyses. All clinical analyses were conducted using GraphPad Prism® 9.3.1 software (San Diego, CA, USA) and JMP® 13 (SAS Institute Inc., Cary, NC, USA).

### Biospecimen collection

All the fresh biospecimens from patients with BLCA were taken by the tumor resection with either radical cystectomy or transurethral resection performed at Osaka Medical and Pharmaceutical University hospital and were immediately stored in the RNAlater reagent (Thermo Fisher Scientific). The inclusion criterion for biospecimen collection mandated a visible papillary/non-papillary tumor of a size exceeding 2 cm. No randomization or blinding was done in the data collection or analyses. Subsequently, all Hematoxylin and Eosin (H&E) stained specimens underwent a rigorous review by a board-certified pathologist to ascertain their histological consistency with BLCA; specimens deviating from this criterion were excluded. For the purposes of this study, we necessitated that tumor sections comprise an average of 60% tumor cell nuclei and exhibit no more than 20% necrosis. The extraction of nucleic acids from both tumor and adjacent normal tissue specimens was facilitated using the DNA/RNA AllPrep kit (QIAGEN). We employed the NanoDrop Microvolume UV–Vis Spectrophotometer (Thermo Fisher Scientific) for the precise quantification of nucleic acids. Furthermore, RNA integrity was assessed using the Agilent 2100 Bioanalyzer (Agilent) to obtain an RNA Integrity Number (RIN); samples presenting a RIN below 7.0 were categorically excluded from this study.

### Whole exome sequencing (WES) and RNA sequencing

In the present study, all the procedures for the library preparation were handled by Maholo LabDroids (humanoid robotic crowd biology) at Robotic Biology Institute Inc. (Tokyo, Japan) [25]. With regard to the RNA-seq library preparation, NEBNext rRNA Depletion Kit was used for the rRNA depletion, followed by the library amplification using NEBNext Ultra II Directional RNA Library Prep Kit for Illumina (New England Biolabs) according to manufacturers’ protocol. For the WES library, the exome region was captured using the xGen® series with Exome Research Panel v1.0 (Integrated DNA Technologies), and libraries were generated using KAPA Hyper plus kit (KAPA Biosystems) according to the manufacturer's protocol. All WES and RNA sequencing were performed on the Illumina NovaSeq 6000 platform using a paired-end flow cell configuration (2 × 150 bp for WES and 2 × 100 bp for RNA sequencing) to ensure a minimum depth of 200 × for WES and 50 million base reads for RNA sequencing."

### Bioinformatic analysis

All analyses were performed using the super-computing resource provided by SHIROKANE (https://gc.hgc.jp/) (Human Genome Center, the Institute of Medical Science, the University of Tokyo).

For the WES analysis, variant calling was conducted through GenomonPipeline:2.6.3 (https://github.com/Genomon-Project/GenomonPipeline). the pipeline continuously invoked the following programs. bwa:0.7.8 (https://github.com/lh3/bwa) was used for the mapping of FASTQ files against GRCh38 with BWA-MEM algorithm. Mutations were determined from BAM files using GenomonFisher.:0.2.1 (https://github.com/Genomon-Project/GenomonFisher). The software included in GenomonPipeline was used not only to analyze cancer samples, but also to compare tumor samples, for which paired normal sample were available, in pairs with normal samples to detect somatic mutations more accurately. In addition, false-positive somatic mutations from cancer genome sequencing data were filtered by Genomon Mutation Filter (https://github.com/Genomon-Project/GenomonMutationFilter). Finally, annotating process for the filtered mutations list was organized by Genomon Mutation Annotator (https://github.com/Genomon-Project/GenomonMutationAnnotator) and ANNOVAR:20,210,202 [26]. These annotations included information on amino acid changes and allele frequencies in several public databases. For publicly available dataset of germline whole-genome sequencing, gnomADv3.0 (https://gnomad.broadinstitute.org/news/2019-10-gnomad-v3-0/) [10] and jMorp-14KJPN (https://jmorp.megabank.tohoku.ac.jp/202112/) [11] were utilized to filter out false positive somatic mutations in the analysis of single nucleotide polymorphisms (SNPs). The mutation landscape was visualized using a script based on CoMut library(https://github.com/vanallenlab/comut). Lolipop mutation plots were generated by using Matplotlib. The script also acquired domain and motif features from UniProt database via Proteins API. <  < # The proteins API: accessing key integrated protein and genome information (10.1093/nar/gkx237)> >.

For the RNA-seq analysis, STAR:2.5.2a (https://github.com/alexdobin/STAR) was used for the mapping of FASTQ on GRCh38. Then, featureCounts (SUBREAD): 2.0.1 (http://subread.sourceforge.net/) was adopted to count the number of reads mapped on exon regions by gene symbol. Raw read counts were used after normalization to TPM (transcripts per million). Regarding the molecular subtypes, we utilized the consensus MIBC subtype (https://github.com/cit-bioinfo/consensusMIBC) [6], the UROMOL subtype (https://github.com/sialindskrog/classifyNMIBC) [13], and the Baylor college subtype [14]. The detection and visualization process of FGFR3-fusion transcript was performed using Arriba platform (https://github.com/suhrig/arriba). Estimated immune-related cell composition was calculated by CIBERSORT [27]. For the deconvolution of tumor microenvironment from the bulk RNA-seq data, EcoTyper (https://github.com/digitalcytometry/ecotyper) was adopted [16]. For differentially expressed gene (DEG) analysis, the DEseq2 platform (https://lashlock.github.io/compbio/R_presentation.html) was conducted using a raw read count matrix from the present cohort.

For publicly available datasets, TCGA data set was analyzed using the cBio Cancer Genomics Portal (cBioPortal; www.cbioportal.org). The raw data from the IMvigor210 trial [17] was downloaded from (http://research-pub.gene.com/IMvigor210CoreBiologies/). Heatmaps were created using Morpheus (https://software.broadinstitute.org/morpheus/).

### Immunohistochemistry

PD-L1 protein expression in immunohistochemistry (IHC) was evaluated in tumor samples obtained from patients using the PD-L1 IHC 22C3 pharmDx assay (Agilent Technologies, Santa Clara, CA) and the 22C3 anti–PD-L1 antibody (Merck & Co., Kenilworth, NJ) [28]. The Combined Positive Score (CPS) method was employed to determine PD-L1 protein expression. This approach quantifies the number of PD-L1 staining cells (tumor cells, lymphocytes, macrophages) and divides it by the total number of viable tumor cells, then multiplies the result by 100. Immunohistochemical staining was conducted using the Discovery ULTRA System (Roche Diagnostics, Basel, Switzerland) as per the manufacturer's guidelines. A panel of antibodies was employed to evaluate the immune profile of the tumor samples, including TIM-3 (rabbit monoclonal antibody, D5D5R, Cell Signaling Technology, Danvers, MA, USA; diluted 1:200), CD8 (monoclonal mouse clone, C8/144B, DAKO; diluted 1:200), and FOXP3 (mouse monoclonal clone, 236A/E7, Abcam; diluted 1:100). At least two researchers independently assessed the immunohistochemistry results to ensure accuracy and reproducibility. The criteria for determining positive cell count were as follows: membrane staining of any intensity for TIM-3 and CD8, or nuclear staining for FOXP3 on ≥ 1% of cells at a high-power field. In the clinical samples, FGFR3 protein expression was evaluated using an FGFR3 rabbit monoclonal antibody (MA5-32,620, ThermoFisher Scientific). The H-score, ranging from 0 to 300, was calculated as (3 × percentage of strongly staining nuclei + 2 × percentage of moderately staining nuclei + percentage of weakly staining nuclei), allowing for a semi-quantitative assessment of protein expression levels. The CPS of PD-L1 and H-score of FGFR3 were evaluated by two board-certified pathologists to provide a robust and reliable foundation for further data analysis and interpretation in the context of molecular pathology.

## Conclusions

We comprehensively investigated the biological implication of aFGFR3 in BLCA. Differential pathways were affected by aFGFR3 between NMIBC and MIBC, particularly emphasizing the significant upregulation of both luminal and basal markers in MIBC/aFGFR3 cases. Crucially, our study underscores the heterogeneous nature of the TME within MIBC/aFGFR3, leading to differential treatment outcomes for CPIs. In particular, favorable ORR in LumP/aFGFR3 and poor ORR in LumP/iFGFR3 were noted. We propose TIM3 for iFGFR3 (ORR: 20% in our cohort) and several immune checkpoint genes for LumP/iFGFR3 (ORR: 5% in our cohort), including IDO1, CCL24, IL1RL1, LGALS4, and NCAM (CD56) as potential druggable targets. These findings offer promising avenues for future precision immunotherapy, indicating a plausible direction for enhancing treatment outcomes in BLCA patients.

### Supplementary Information

**Additional file 1: Supplementary Figure 1.** (A) Kaplan–Meier curves for overall survival (OS) in non-muscle invasive bladder cancer (NMIBC) (upper panel: 124 patients) and muscle-invasive bladder cancer (MIBC) (lower panel: 265 patients). (B) Violin plots for mRNA expression levels (TPM: transcripts per million) of FGFR families (FGFR1-4) in normal (*n*=35) and tumor (*n*=389) tissues. (C) Violin plots for mRNA expression levels (TPM) in each FGFR family in 389 tumor samples according to the presence or absence of mutation. The difference was assessed by the Mann–Whitney U test. (D) Comparison of single nucleotide polymorphisms (SNPs) between GnomADv3.0, an integrative germline dataset of 71,702 individuals (mostly Western population) [10] and Japanese germline dataset (jMorp-14KJPN) of 28,258 allele number [11] within the *FGFR3* gene locus (NM_000142). (E) FGFR3 mRNA expression levels in *FGFR3* intact, *FGFR3* SNPs, FGFR3 mutants in 389 bladder cancer (BLCA) patients (The difference in the expression level was assessed by the Mann–Whitney U test; *p<0.05, n.s: non-significant). (F) Kaplan–Meier curves for cancer-specific survival (CSS), OS and progression-free survival (PFS) in NMIBC (left panels: 124 patients) and MIBC (right panels: 265 patients) according to the *FGFR3* genetic alternations. Log-rank test was utilized to examine the difference in survival.

** Additional file 2: Supplementary Figure 2.** (A) Mutation plot of FGFR3 (NM_000142) in 408 TCGA bladder cancer cohort [9]. (B) Mutation plots of FGFR3 (NM_000142) for cases with NMIBC (*n*=124) and MIBC (*n*=265). (C) Kaplan-Meier curves for OS in MIBC (*n*=265) according to mutations at the kinase domain (KD). (D) Estimated proportion of mutations at KD in NMIBC and MIBC cases. (E) FGFR3 mRNA expression level among the nonsynonymous mutations and SNPs in 389 BLCA patients. (F) FGFR3 mRNA expression levels according to *FGFR3* alterations in 389 BLCA samples. Four samples harboring both mutation and fusion were assigned to the fusion group. The difference in the FGFR3 mRNA was assessed by the Mann–Whitney U test (*p<0.05). (G) Pie charts of the H-score for FGFR3 according to *FGFR3* status. Chi-square test was utilized to assess the difference. (H) Representative images of Immunohistochemistry for FGFR3. H-score was evaluated by (3 x percentage of strongly staining nuclei + 2 x percentage of moderately staining nuclei + percentage of weakly staining nuclei, giving a range of 0 to 300).

** Additional file 3: Supplementary Figure 3.** (A) Hierarchical clustering for the 18 tumor differentiation classifier genes from Baylor College which define the two subgroups with distinct expression patterns [14]. (B) Estimated proportion of consensus MIBC subtypes [6] in NMIBC and MIBC cases. (C) Estimated proportion of UROMOL NMIBC subtypes [13] in NMIBC and MIBC cases. (D) Estimated proportion of Baylor college subtypes in NMIBC and MIBC cases. (E) Pie charts of *FGFR3* alterations in each molecular subtype. Fisher’s exact test was utilized to assess the difference. (F) FGFR3 mRNA expression levels (transcripts per million: TPM) according to the Baylor college subtypes. The difference was assessed by the Mann–Whitney U test. (G) Gene set enrichment analysis in 389 BLCA (iFGFR3: 295 cases vs aFGFR3: 94 cases) plotting all human MSigDB collections (Hallmark, C1-8: 23734 gene sets) by false discovery rate q-value (FDR-q) and normalized enrichment score (NES). (H) Gene set enrichment analysis (GSEA) of “HALLMARK_EPITHELIAL_MESENCHYMAL_TRANSITION”, and“HALLMARK_ALLOGRAFT_REJECTION” that were top 2 downregulated pathways in aFGFR3.

** Additional file 4: Supplementary Figure 4.** (A) Representative images of immunohistochemistry for PD-L1 using the PD-L1 IHC 22C3 pharmDx assay (Agilent Technologies, Santa Clara, CA) and the 22C3 anti–PD-L1 antibody (Merck & Co., Kenilworth, NJ) [28]. The PD-L1 protein expression is determined by the Combined Positive Score (CPS), the number of PD-L1 staining cells (tumor cells, lymphocytes, macrophages) divided by the total number of viable tumor cells multiplied by 100. Corresponding hematoxylin-eosin stain (HE stain) is shown in the upper series. (B) CD274 mRNA expression level according to the CPS. (C) Pie chart of the PD-L1 CPS score (364 of 389 tumors were evaluable). (D) Pie chart of the PD-L1 CPS score in aFGFR3 (*n*=84) and iFGFR3 (*n*=280). (E) Representative images of immunohistochemistry for CD8. The cell count was evaluated at 400x magnification. (F) Cell count of CD8+ cells for the stromal, intratumor, and total region with high power field (x400). Mann-Whitney U test was used to examine the difference. (G) Heatmap of putative immune checkpoint genes according to *FGFR3* status. (H) mRNA expression (transcripts per million: TPM) of immune checkpoint genes between iFGFR3 (*n*=295) and aFGFR3 (*n*=94) (Mann-Whitney U test was used to examine the difference. * *p*<0.05, **p<0.01, *** *p*<0.001, **** *p*<0.0001).

** Additional file 5: Supplementary Figure 5.** (A) Correlation between “estimated proportion of T-cell CD8 from CIBERSORTx” and “cell count of CD8+ positive cells in HPF” (left panel), and “T-cells regulatory” and cell count of FOXP3 positive cells in HPF” (right panel), respectively. (B,C) The estimated proportion of each immune cell type from CIBERSORTx comparing iFGFR3 and aFGFR3 in (B) NMIBC and (C) MIBC. Mann-Whitney U test was used to examine the difference. * *p*<0.05,** *p*<0.001, *** *p*<0.0001, n.s: non-significant. (D) Pearson correlation coefficient among the estimated proportion of immune-related cells in NMIBC/iFGFR3 (*n*=69) and NMIBC/aFGFR3 (*n*=55). (E) Pearson correlation coefficient among the estimated proportion of immune-related cells in MIBC/iFGFR3 (*n*=226) and MIBC/aFGFR3 (*n*=39). (F) Individual cell states in each of ten cell types from the EcoTyper analysis. Representative genes defining cell states are shown in each cell type.

** Additional file 6: Supplementary Figure 6.** (A) Estimated proportion of treatment response to CPIs including pembrolizumab (*n*=60) and avelumab (*n*=12). (B) Estimated proportion of treatment response to CPIs among cellular ecotypes (CEs) defined by EcoTyper. (C) Correlation of tumor mutation burden (TMB) with PD-L1 combined positive score (CPS), TIM3 positive cell count in high power field, and FGFR3 mRNA expression level. (D) Estimated proportion of consensus MIBC subtypes among in IMvigor210 trial [17] (*n*=274). (E) The ORR in the IMvigor210 trial (*n*=274) and the present cohort (OMPU: *n*=72) treated with CPIs in LumU subtype (left panel) and other subtypes including LumNS, NE-like, and stromal-rich (right panel). Fisher’s exact test was performed to assess the difference of the ORR according to *FGFR3* status. Note that the data from IMvigor210 does not include the information on *FGFR3* fusions.

** Additional file 7: Supplementary Table 1.**

** Additional file 8:  Supplementary Table 2.**

** Additional file 9:  Supplementary Table 3.**

** Additional file 10:  Supplementary Table 4.**

** Additional file 11:  Supplementary Table 5.**

## Data Availability

The raw sequencing data and clinical data can be obtained from the Osaka Medical and Pharmaceutical University Translational Research Program Biobank (OMPU-TR Biobank) (https://www.ompu.ac.jp/department/rdcenter/transregular/); however, restrictions apply to the availability of these data, and they are not publicly accessible. Data are available to National Cancer Center Research Institute and OMPU investigators and their external affiliates, including academic and commercial partners, provided that they have approval from the Institutional Review Board (IRB) and a data use agreement. Samples and data shared with external entities must be de-identified. Any additional information required to reanalyze the data reported in this work is available from the Lead Contact (KK; kazumasa.komura@ompu.ac.jp, AY; ayoshimi@ncc.go.jp) upon request.

## Abbreviations

BLCA: Bladder cancer
CE: Cellular ecotypes
CI: Confidence interval
CPIs: Checkpoint inhibitors
CPS: Combined positive score
CR: Complete response
CSS: Cancer-specific survival
EMT: Epithelial-mesenchymal transition
FGFR3: Fibroblast growth factor receptor 3
GSEA: Gene set enrichment analysis
KD: Kinase domain
MIBC: Muscle invasive bladder cancer
NMIBC: Non-muscle invasive bladder cancer
ORR: Objective response rate
OS: Overall survival
PD: Progressive disease
PFS: Progression-free survival
PR: Partial response
SD: Stable disease
SNPs: Single nucleotide polymorphisms
TMB: Tumor mutation burden
TME: Tumor microenvironment
UC: Urothelial carcinoma
WES: Whole exome sequencing
