# Supplementary figures and images for: The Impact of FGFR3 Alterations on the Tumor Microenvironment and the Efficacy of Immune Checkpoint Inhibitors in Bladder Cancer
Source: Mol Cancer. 2023 Nov 18;22:185. doi: 10.1186/s12943-023-01897-6 (PMC10657138; doi:10.1186/s12943-023-01897-6)

Supplementary Figure 1

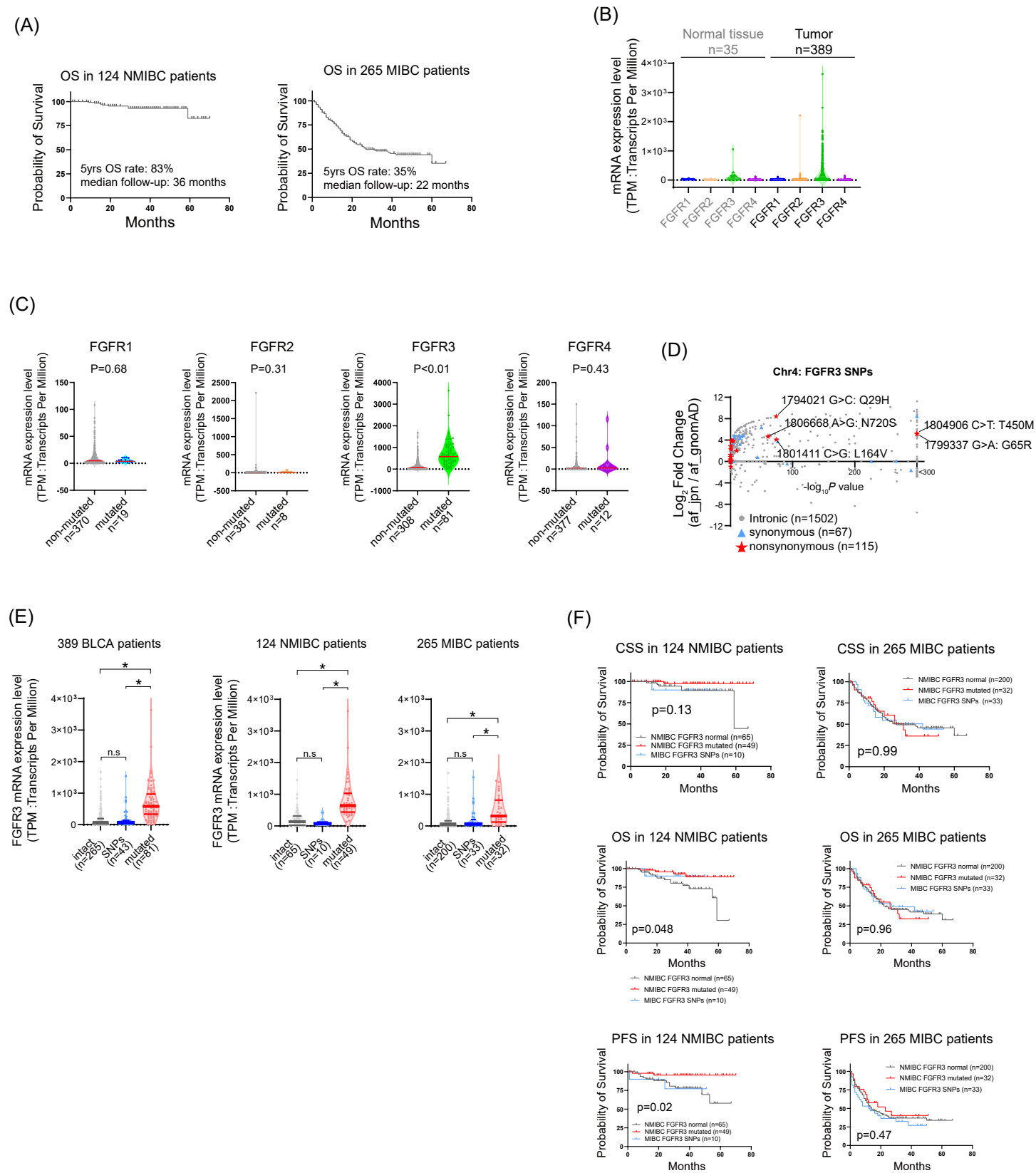

Supplement: Supplementary file 1 — Additional file 1: Supplementary Figure 1. (A) Kaplan–Meier curves for overall survival (OS) in non-muscle invasive bladder cancer (NMIBC) (upper panel: 124 patients) and muscle-invasive bladder cancer (MIBC) (lower panel: 265 patients). (B) Violin plots for mRNA expression levels (TPM: transcripts per million) of FGFR families (FGFR1-4) in normal (n=35) and tumor (n=389) tissues. (C) Violin plots for mRNA expression levels (TPM) in each FGFR family in 389 tumor samples according to the presence or absence of mutation. The difference was assessed by the Mann–Whitney U test. (D) Comparison of single nucleotide polymorphisms (SNPs) between GnomADv3.0, an integrative germline dataset of 71,702 individuals (mostly Western population) [10] and Japanese germline dataset (jMorp-14KJPN) of 28,258 allele number [11] within the FGFR3 gene locus (NM_000142). (E) FGFR3 mRNA expression levels in FGFR3 intact, FGFR3 SNPs, FGFR3 mutants in 389 bladder cancer (BLCA) patients (The difference in the expression level was assessed by the Mann–Whitney U test; *p<0.05, n.s: non-significant). (F) Kaplan–Meier curves for cancer-specific survival (CSS), OS and progression-free survival (PFS) in NMIBC (left panels: 124 patients) and MIBC (right panels: 265 patients) according to the FGFR3 genetic alternations. Log-rank test was utilized to examine the difference in survival. [file 12943_2023_1897_MOESM1_ESM.pdf]

Supplementary Figure 2

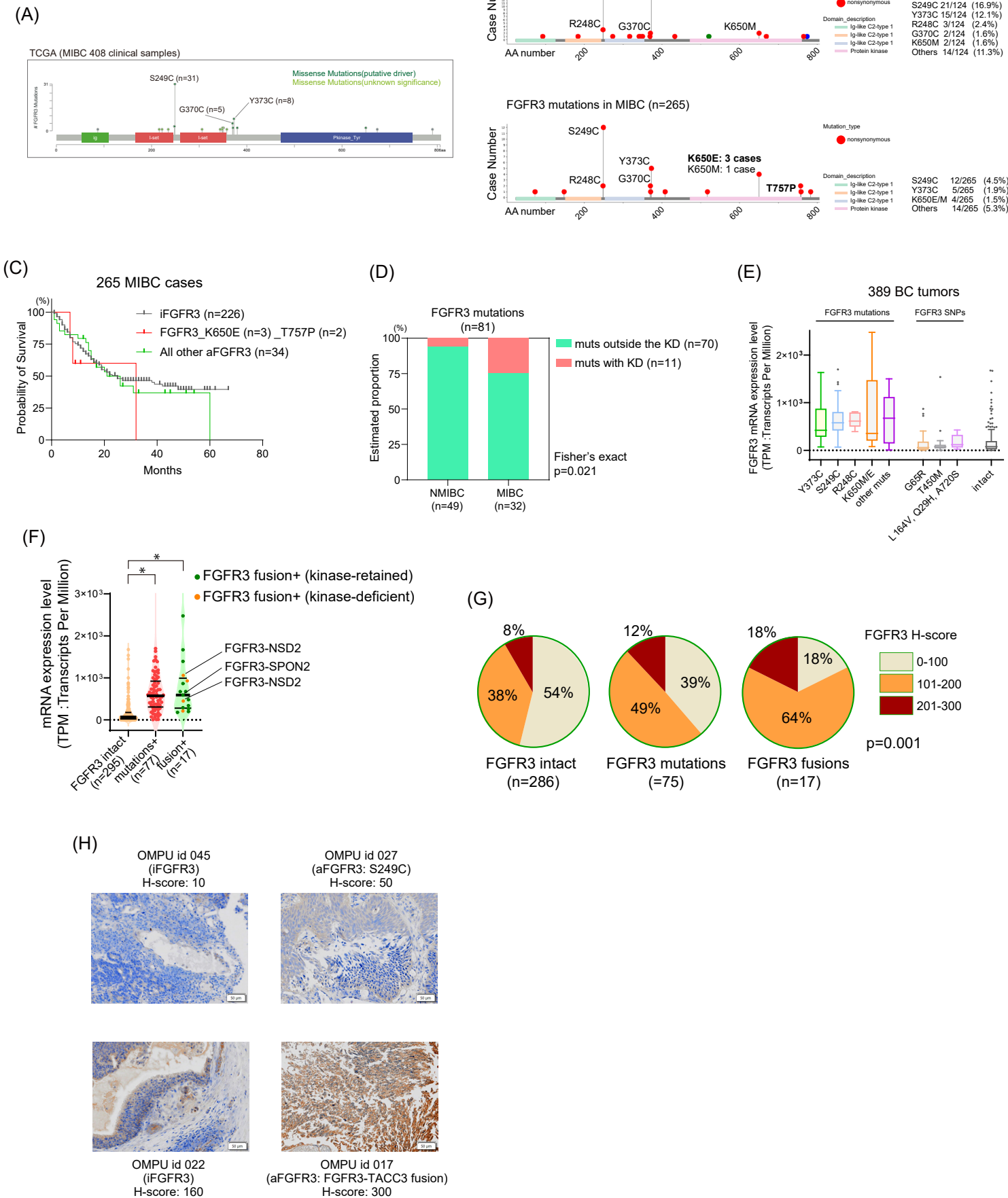

Supplement: Supplementary file 2 — Additional file 2: Supplementary Figure 2. (A) Mutation plot of FGFR3 (NM_000142) in 408 TCGA bladder cancer cohort [9]. (B) Mutation plots of FGFR3 (NM_000142) for cases with NMIBC (n=124) and MIBC (n=265). (C) Kaplan-Meier curves for OS in MIBC (n=265) according to mutations at the kinase domain (KD). (D) Estimated proportion of mutations at KD in NMIBC and MIBC cases. (E) FGFR3 mRNA expression level among the nonsynonymous mutations and SNPs in 389 BLCA patients. (F) FGFR3 mRNA expression levels according to FGFR3 alterations in 389 BLCA samples. Four samples harboring both mutation and fusion were assigned to the fusion group. The difference in the FGFR3 mRNA was assessed by the Mann–Whitney U test (*p<0.05). (G) Pie charts of the H-score for FGFR3 according to FGFR3 status. Chi-square test was utilized to assess the difference. (H) Representative images of Immunohistochemistry for FGFR3. H-score was evaluated by (3 x percentage of strongly staining nuclei + 2 x percentage of moderately staining nuclei + percentage of weakly staining nuclei, giving a range of 0 to 300). [file 12943_2023_1897_MOESM2_ESM.pdf]

Supplementary Figure 3

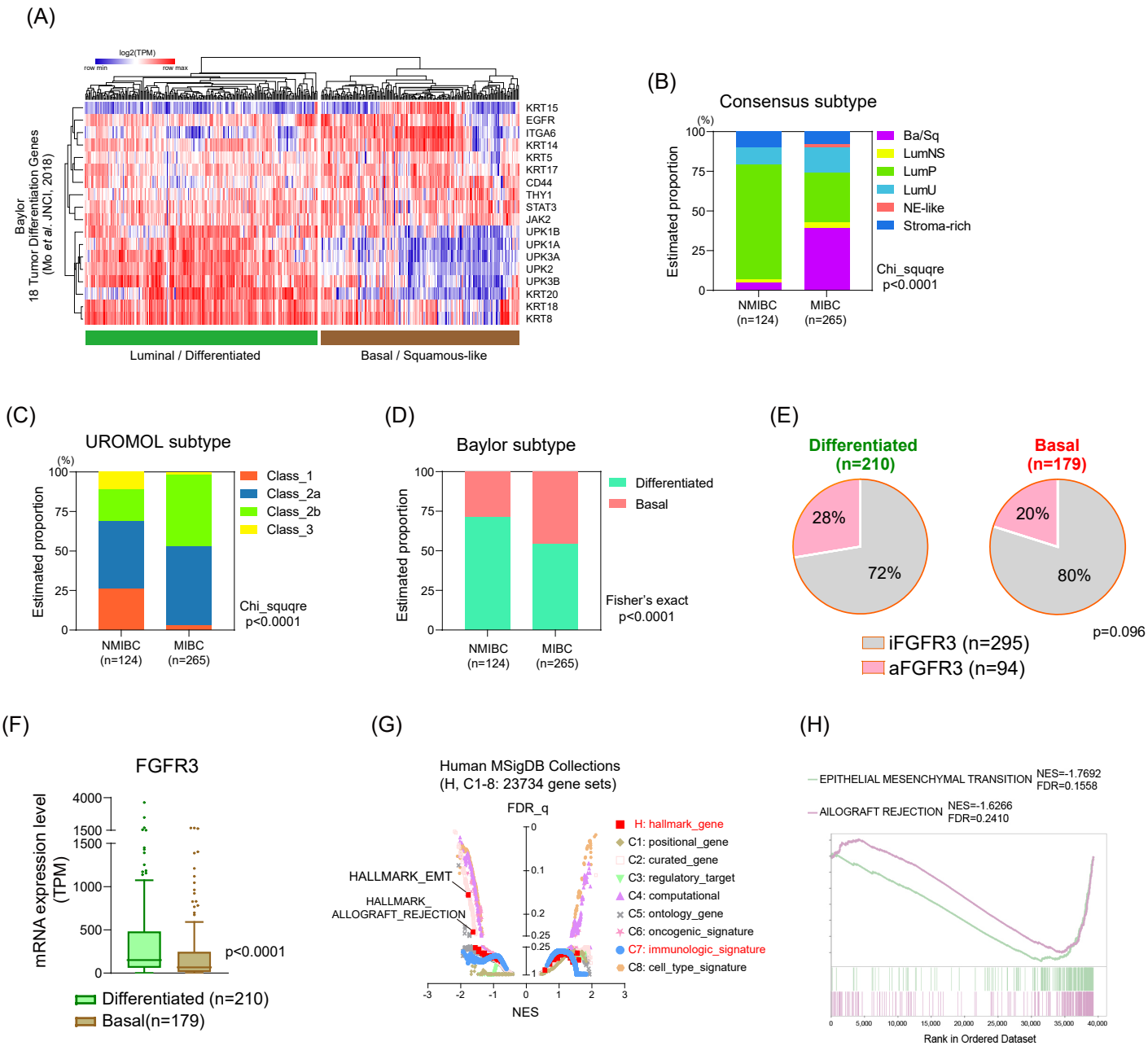

Supplement: Supplementary file 3 — Additional file 3: Supplementary Figure 3. (A) Hierarchical clustering for the 18 tumor differentiation classifier genes from Baylor College which define the two subgroups with distinct expression patterns [14]. (B) Estimated proportion of consensus MIBC subtypes [6] in NMIBC and MIBC cases. (C) Estimated proportion of UROMOL NMIBC subtypes [13] in NMIBC and MIBC cases. (D) Estimated proportion of Baylor college subtypes in NMIBC and MIBC cases. (E) Pie charts of FGFR3 alterations in each molecular subtype. Fisher’s exact test was utilized to assess the difference. (F) FGFR3 mRNA expression levels (transcripts per million: TPM) according to the Baylor college subtypes. The difference was assessed by the Mann–Whitney U test. (G) Gene set enrichment analysis in 389 BLCA (iFGFR3: 295 cases vs aFGFR3: 94 cases) plotting all human MSigDB collections (Hallmark, C1-8: 23734 gene sets) by false discovery rate q-value (FDR-q) and normalized enrichment score (NES). (H) Gene set enrichment analysis (GSEA) of “HALLMARK_EPITHELIAL_MESENCHYMAL_TRANSITION”, and“HALLMARK_ALLOGRAFT_REJECTION” that were top 2 downregulated pathways in aFGFR3. [file 12943_2023_1897_MOESM3_ESM.pdf]

Supplementary Figure 4

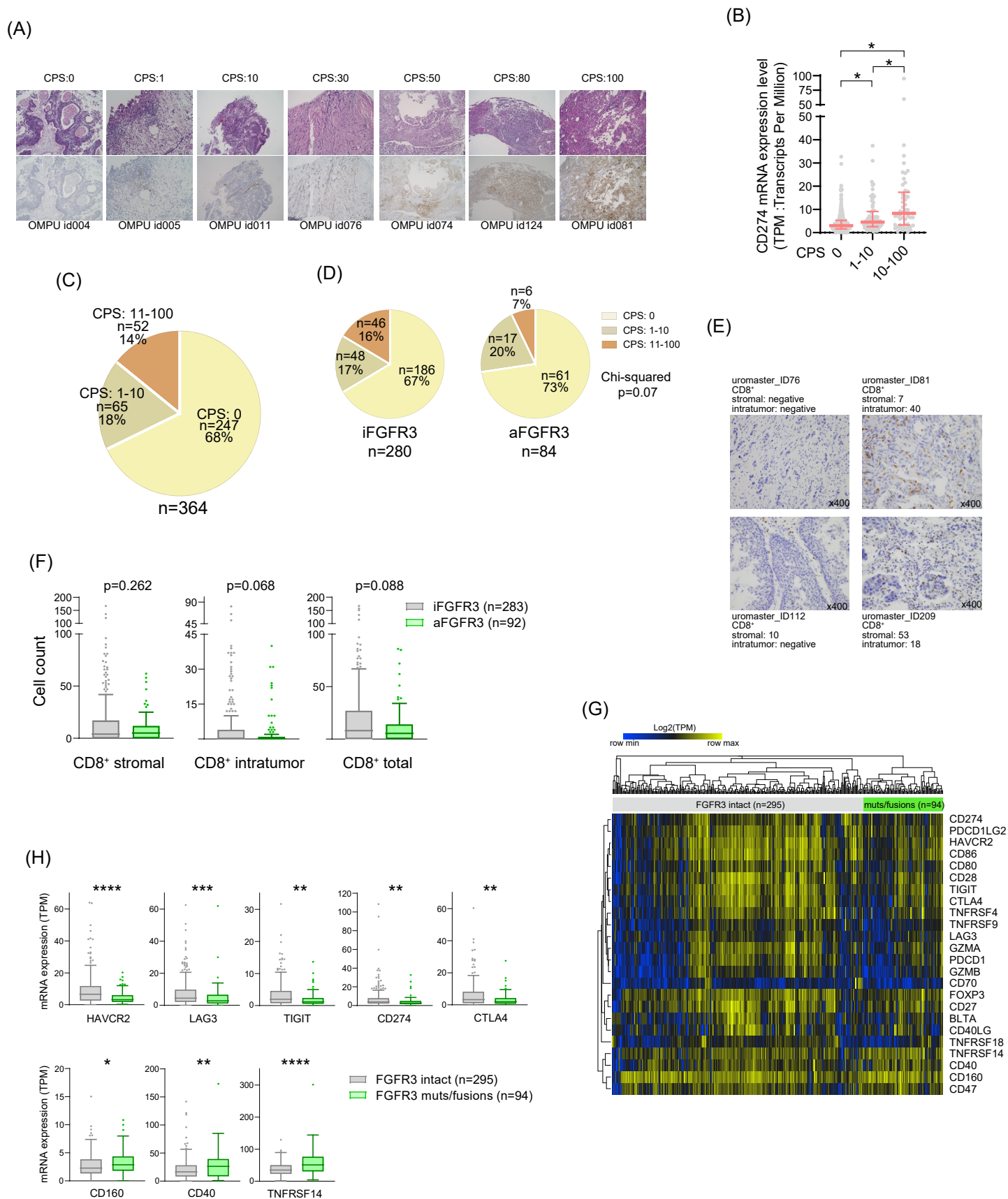

Supplement: Supplementary file 4 — Additional file 4: Supplementary Figure 4. (A) Representative images of immunohistochemistry for PD-L1 using the PD-L1 IHC 22C3 pharmDx assay (Agilent Technologies, Santa Clara, CA) and the 22C3 anti–PD-L1 antibody (Merck & Co., Kenilworth, NJ) [28]. The PD-L1 protein expression is determined by the Combined Positive Score (CPS), the number of PD-L1 staining cells (tumor cells, lymphocytes, macrophages) divided by the total number of viable tumor cells multiplied by 100. Corresponding hematoxylin-eosin stain (HE stain) is shown in the upper series. (B) CD274 mRNA expression level according to the CPS. (C) Pie chart of the PD-L1 CPS score (364 of 389 tumors were evaluable). (D) Pie chart of the PD-L1 CPS score in aFGFR3 (n=84) and iFGFR3 (n=280). (E) Representative images of immunohistochemistry for CD8. The cell count was evaluated at 400x magnification. (F) Cell count of CD8+ cells for the stromal, intratumor, and total region with high power field (x400). Mann-Whitney U test was used to examine the difference. (G) Heatmap of putative immune checkpoint genes according to FGFR3 status. (H) mRNA expression (transcripts per million: TPM) of immune checkpoint genes between iFGFR3 (n=295) and aFGFR3 (n=94) (Mann-Whitney U test was used to examine the difference. * p<0.05, **p<0.01, *** p<0.001, **** p<0.0001). [file 12943_2023_1897_MOESM4_ESM.pdf]

# Supplementary Figure 5

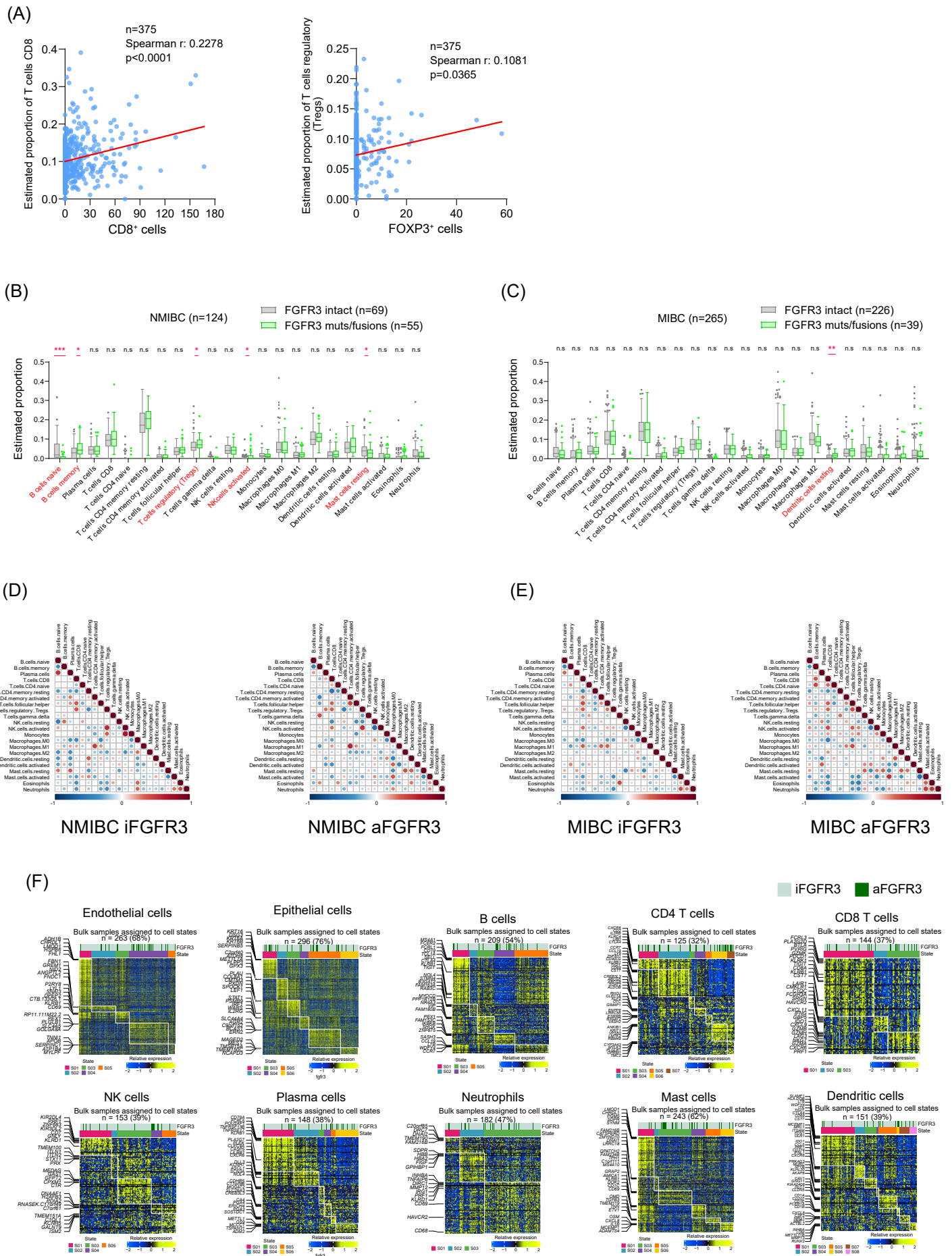

Supplement: Supplementary file 5 — Additional file 5: Supplementary Figure 5. (A) Correlation between “estimated proportion of T-cell CD8 from CIBERSORTx” and “cell count of CD8+ positive cells in HPF” (left panel), and “T-cells regulatory” and cell count of FOXP3 positive cells in HPF” (right panel), respectively. (B,C) The estimated proportion of each immune cell type from CIBERSORTx comparing iFGFR3 and aFGFR3 in (B) NMIBC and (C) MIBC. Mann-Whitney U test was used to examine the difference. * p<0.05,** p<0.001, *** p<0.0001, n.s: non-significant. (D) Pearson correlation coefficient among the estimated proportion of immune-related cells in NMIBC/iFGFR3 (n=69) and NMIBC/aFGFR3 (n=55). (E) Pearson correlation coefficient among the estimated proportion of immune-related cells in MIBC/iFGFR3 (n=226) and MIBC/aFGFR3 (n=39). (F) Individual cell states in each of ten cell types from the EcoTyper analysis. Representative genes defining cell states are shown in each cell type. [file 12943_2023_1897_MOESM5_ESM.pdf]

Supplementary Figure 6

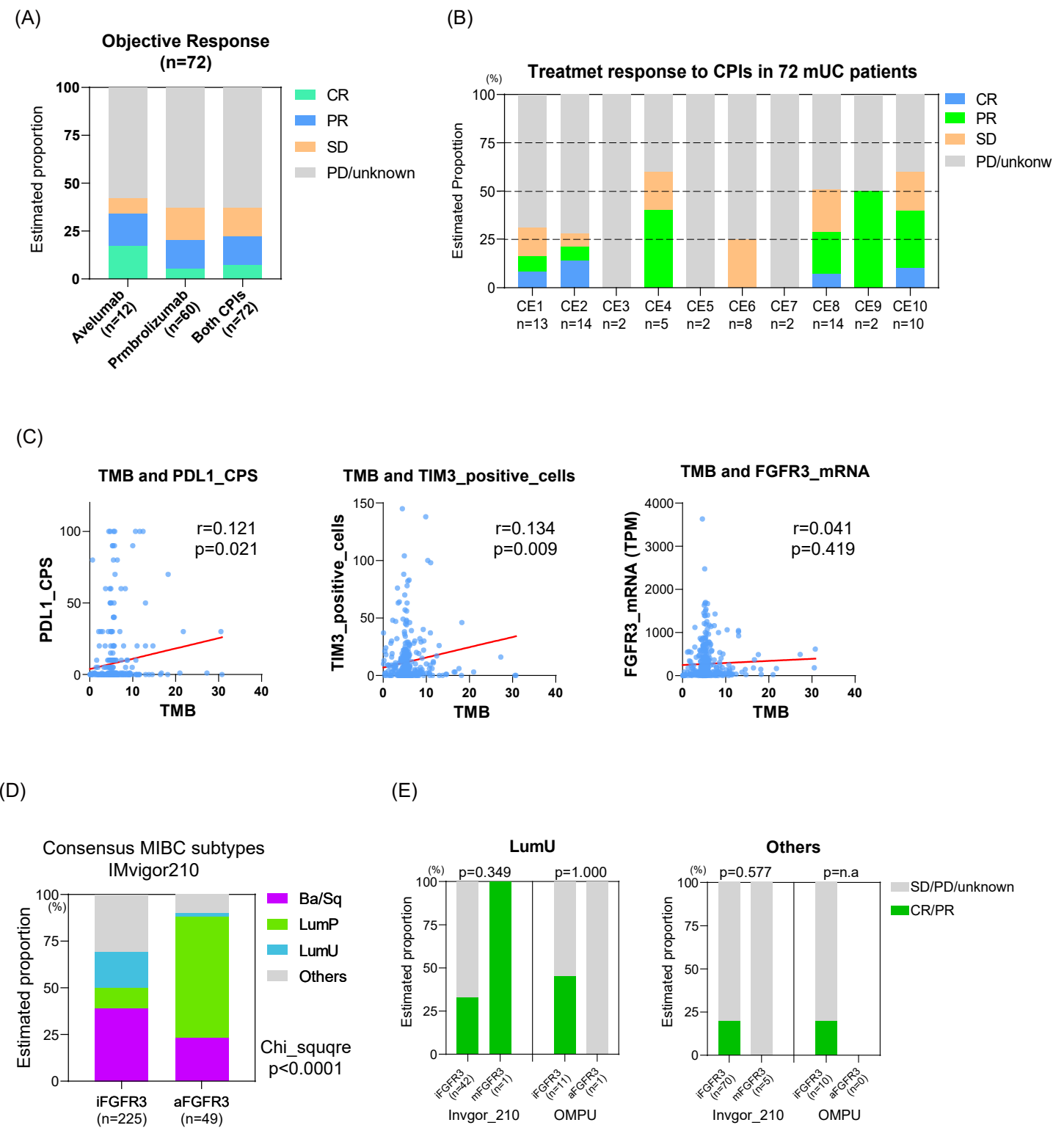

Supplement: Supplementary file 6 — Additional file 6: Supplementary Figure 6. (A) Estimated proportion of treatment response to CPIs including pembrolizumab (n=60) and avelumab (n=12). (B) Estimated proportion of treatment response to CPIs among cellular ecotypes (CEs) defined by EcoTyper. (C) Correlation of tumor mutation burden (TMB) with PD-L1 combined positive score (CPS), TIM3 positive cell count in high power field, and FGFR3 mRNA expression level. (D) Estimated proportion of consensus MIBC subtypes among in IMvigor210 trial [17] (n=274). (E) The ORR in the IMvigor210 trial (n=274) and the present cohort (OMPU: n=72) treated with CPIs in LumU subtype (left panel) and other subtypes including LumNS, NE-like, and stromal-rich (right panel). Fisher’s exact test was performed to assess the difference of the ORR according to FGFR3 status. Note that the data from IMvigor210 does not include the information on FGFR3 fusions. [file 12943_2023_1897_MOESM6_ESM.pdf]
